# 

**Published:** 1868-03

**Authors:** 


### CONTENTS.

ORIGINAL COMMUNICATIONS.

Valedictory address on behalf of the graduating class at the Twenty-Second Annual Commencement of the Ohio Dental College .......... 93
On the Physiological action of the muscles concerned in the movements of the Lower Jaw .......... 103
The Rubber Suits .......... 110
Keeping the Teeth dry .......... 121

SELECTIONS.

Nitrous Oxide .......... 124
Cheiloplasty .......... 125
Medical Education .......... 125
Ozone .......... 127
Importance of Ventilation .......... 129
Illinois State Dental Society .......... 130
Northern Ohio Dental Association .......... 130

EDITORIAL.

A Change—the Result .......... 131
Ohio Dental College .......... 132
Obituary—Dr. John P. Wilson .......... 133
Obituary—Dr. Robert Nelson .......... 134

